# Impact of at-home circuit training on cardiovascular risk, functional capacity and quality of life in older adults with knee osteoarthritis and diabesity: a randomized controlled trial

**DOI:** 10.3934/publichealth.2026018

**Published:** 2026-03-09

**Authors:** Sameer Badri Al-Mhanna, Zahid Nori Kamaluddin, Georgian Badicu, Wan Syaheedah Wan Ghazali, Mahaneem Mohamed, Ayu Suzailiana Muhamad, Monira I. Aldhahi, Shaifuzain Ab Rahman, Hafeez Abiola Afolabi, Ab Hamid Siti-Azrin, Maha H. Alhussain, Fatma Hilal Yagin, Mehmet Gülü, Abubakar Ibrahim, Clemens Drenowatz, Alexios Batrakoulis

**Affiliations:** 1 Department of Physiology, Saveetha Medical College and Hospital, Saveetha Institute of Medical and Technical Sciences, Chennai, Tamil Nadu, India; 2 Department of Higher Studies, Al-Qasim Green University, Babylon, Iraq; 3 Department of Physical Education and Special Motricity, Transilvania University of Braşov, Braşov, Romania; 4 Department of Physiology, School of Medical Sciences, Universiti Sains Malaysia, Kubang Kerian 16150, Kelantan, Malaysia; 5 Exercise and Sports Science Program, School of Health Sciences, Universiti Sains Malaysia, Kubang Kerian 16150, Kelantan, Malaysia; 6 Department of Rehabilitation Sciences, College of Health and Rehabilitation Sciences, Princess Nourah bint Abdulrahman University, Riyadh 11671, Saudi Arabia; 7 Department of Orthopedic, Universiti Sains Malaysia, Kubang Kerian 16150, Kelantan, Malaysia; 8 Department of General Surgery, School of Medical Sciences, Universiti Sains Malaysia, Kubang Kerian 16150, Kelantan, Malaysia; 9 Biostatistics and Research Methodology Unit, School of Medical Sciences, Universiti Sains Malaysia, Kubang Kerian 16150, Kelantan, Malaysia; 10 Department of Food Science and Nutrition, College of Food and Agricultural Science, King Saud University, Riyadh 11451, Saudi Arabia; 11 Department of Biostatistics, Faculty of Medicine, Malatya Turgut Ozal University, Malatya 44210, Turkey; 12 Department of Sports Management, Faculty of Sport Sciences, Kirikkale University, Kirikkale, Turkey; 13 School of Medical Sciences University Sains Malaysia, Department of Obstetrics and Gynecology; 14 Division of Sport, Physical Activity and Health, University of Education Upper Austria, Austria; 15 Department of Life Sciences, School of Sciences, European University Cyprus, Nicosia 2404, Cyprus; 16 Department of Physical Education and Sport Science, School of Physical Education, Sport Science and Occupational Therapy, Komotini 69100, Greece

**Keywords:** aerobic exercise, strength training, glycemic control, blood pressure, oxidative stress, metabolic syndrome

## Abstract

**Background:**

The present study was designed to investigate the effectiveness of a 12-week home-based circuit training (HBCT) protocol on numerous indices associated with knee osteoarthritis (KOA) and cardiometabolic health among older adult patients with KOA and the metabolic overlap between obesity and type 2 diabetes, also known as diabesity.

**Methods:**

This randomized controlled trial was registered at the Clinical Trials Registry (Date: March 7, 2024; ID: NCT06309654). Seventy overweight or obese participants (56% male) with a mean age of 62.2 ± 6.1 years were randomly allocated to the exercise group (n = 35, HBCT) or the non-exercising control group (n = 35, CON). HBCT performed a progressive protocol, lasting 20–60 min, three times per week. Each exercise session comprised seven exercises, with 15–30 repetitions, a 1-min passive rest between exercises, and 2–4 rounds per session. The assessment of outcomes included knee injury and osteoarthritis outcome score, cardiometabolic risk factors, cardiorespiratory fitness, and renal function at pre- and post-training.

**Results:**

The results demonstrate that HBCT led to a notable enhancement in the majority of cardiometabolic health indicators (e.g., anthropometrics, blood pressure, and glucose metabolism), KOA parameters related to quality of life and functionality, and cardiorespiratory fitness when compared with CON (p < 0.05). No significant changes were observed in total bilirubin, sodium, urea, and resting heart rate between HBCT and CON.

**Conclusion:**

The present results suggest that an injury-free HBCT program may improve numerous cardiometabolic health- and KOA-related indicators in older adult patients with KOA and diabesity.

## 1. Introduction

The metabolic overlap between obesity and type 2 diabetes, driven by excess adiposity and insulin resistance, has been defined as diabesity [1]. The diabesity epidemic has been documented as a major public health issue, adversely affecting healthcare systems globally due to the association with various chronic diseases [2]. Importantly, adult obesity rates have doubled since 1980, and this global trend has been accompanied by a parallel increase in the prevalence of type 2 diabetes mellitus (T2DM) [3]. According to the World Health Organisation (WHO), in 2016, over 1.9 billion adults were identified as overweight, with a subset of 650 million classified as obese [4]. Meanwhile, osteoarthritis is the most common joint disease worldwide, demonstrating a high rate of comorbidities [5], ranging from 68% to 85% [6]. Cardiovascular disease (CVD), stroke, T2DM, obesity, cognitive impairment, anxiety, and depression are usually linked to osteoarthritis, and the association between osteoarthritis and disability is thought to be attributed to coexisting comorbidities rather than the disease itself [7],[8]. Comorbidity is associated with increased knee pain and a poorer prognosis in older people with osteoarthritis [9]. The risk of knee osteoarthritis (KOA) can be increased by 15% with a unit of increase in body mass index (BMI) [10]. The findings of earlier research studies have indicated an association between obesity and knee osteoarthritis (KOA) in joints that do not bear weight. This association suggests the presence of a systemic mechanism involving inflammatory adipokines and a local risk factor resulting from increased mechanical joint loading due to overweight or obesity [11]. This association may lead to the need for total knee arthroplasty in individuals with a high BMI [12].

Sufficient physical activity levels are critical for people with metabolic and musculoskeletal health impairments from a physiological standpoint. In particular, physical activity and various exercise modes increase muscular fitness and improve physical function [13],[14], as well as lowering inflammatory cytokines and protecting the knee joint [15]. Such positive exercise-induced adaptations have also been observed in several cardiometabolic health-related indicators among patients with diabesity [16]–[21]. Interestingly, lower limb function and aerobic capacity were reported as the optimal exercise strategy for treating patients with KOA [22]. However, Bennell, Hinman [23] suggested that the best approach to address the diverse complications associated with osteoarthritis is combined aerobic and resistance training. Also, Nguyen, Lefevre-Colau [24] underlined the key role of muscle-strengthening activities as a foundational piece of the exercise therapy puzzle among patients with KOA, showing that resistance training can be an effective and safe exercise mode for this population. Such positive adaptations may help patients with diabesity and KOA to limit the detrimental effects of inactivity highly associated with poor metabolic and musculoskeletal health [25]–[28]. Noteworthy, home-based exercise training has been recently reported as an effective solution for inducing health and fitness benefits in older adults who commonly present with KOA and/or cardiometabolic dysregulation [29]–[34].

Also, circuit training appears to be a popular [35] and effective approach for maximizing the advantages of physical exercise, enhancing body composition, cardiorespiratory, and muscular fitness in various populations, including older adults with chronic diseases, including KOA [36],[37]. Combined resistance- and aerobic-based activities sustain an elevated heart rate throughout the workout [38], providing greater benefits compared to traditional aerobic exercise [39],[40]. Additionally, circuit training appears to be a time-efficient approach, providing an engaging exercise solution, increasing adherence [41],[42], improving body composition [43], and enhancing adipose tissue lipolysis to a greater extent compared to traditional aerobic exercise [44]. It is worth mentioning that circuit-based exercise solutions that incorporate a hybrid format demonstrate substantial improvements in several cardiometabolic and musculoskeletal health-related markers among previously inactive overweight and obese people [45]–[49].

Despite beneficial training-induced adaptations, the optimal exercise strategy for populations with diabesity and KOA presents some shortcomings in the current literature. More importantly, there are no explicit exercise recommendations in the current KOA guidelines [49],[50]. Combining several disease treatment regimens is sometimes impossible since one therapy may impact the natural course of a coexisting condition or interact unfavorably with another medication [51]. Also, this particular cohort is prone to experience cardiovascular complications, impaired musculoskeletal health, physical limitations, poor functional capacity, and movement-induced pain. The investigation of home-based circuit training in older adults is justified by age-related declines in muscle strength, balance, and cardiometabolic health, alongside substantial barriers to facility-based exercise, including mobility, financial, and accessibility constraints [52]. As a time-efficient, scalable, and low-cost modality integrating aerobic and resistance components, home-based circuit training may support functional independence and mitigate chronic disease risk in ageing populations, underscoring the need for robust empirical evidence to inform pragmatic and sustainable physical activity strategies [33].

We hypothesized that a 12-week home-based circuit training (HBCT) protocol may be effective in improving various outcomes related to knee osteoarthritis symptoms and cardiometabolic health in previously inactive, older adult patients with KOA and diabesity compared with a non-exercising control group. Therefore, the objective of this pragmatic trial was to investigate the effects of an HBCT protocol on i) anthropometrics, ii) resting cardiovascular function, iii) glycemic control, iv) renal function, v) inflammation, and vi) physical function parameters of previously inactive, older adult patients with KOA and diabesity.

## 2. Methods

### 2.1. Study design

This study constituted a component of a larger trial—the purpose, methodology, and inclusion/exclusion criteria of which have been described in detail in a previous publication [53]. In brief, this randomized controlled trial has been registered at the Clinical Trials Registry (ClinicalTrials.gov; March 7, 2024; ID: NCT06309654) and can be accessed via the following link: https://ichgcp.net/clinical-trials-registry/NCT06309654.

**Figure 1.**
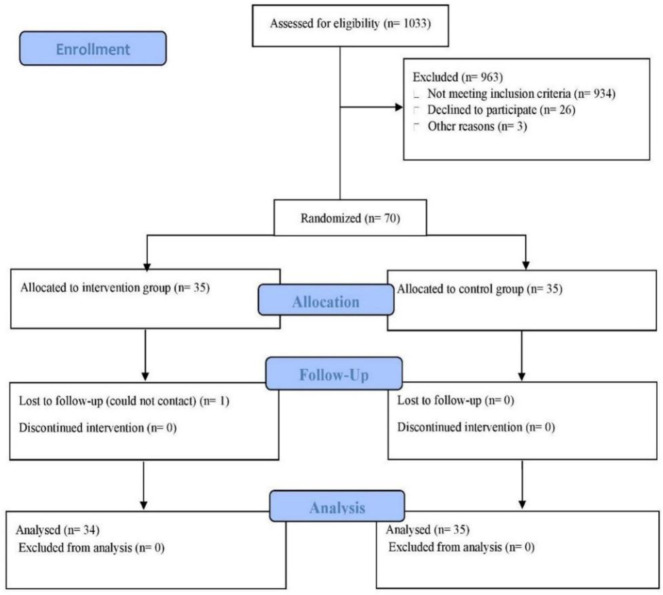
CONSORT flow diagram of the study.

The study was conducted at the Physiology Laboratory of the School of Medical Sciences at the University Science Malaysia. The study was approved by the Human Research Ethics Committee (approval code: JEPeM 21050374) and conforms to the provisions of the Declaration of Helsinki. Written informed consent was obtained from all patients. This study was conducted in accordance with the Consolidated Standards of Reporting Trials (CONSORT) guidelines. A total of 1033 patients with diagnosed KOA were recruited via a poster distributed in the orthopedics clinic at USM Hospital. Prior to the commencement of the study, written informed consent was obtained from all participants who met the inclusions criteria (n = 70). Those who agreed to participate were then randomly assigned to either the CON or HBCT group. Simple randomization (http://www.randomization.com) through computer-generated random allocation sequences was used by an independent statistician. Throughout the process, assessors were blinded and unaware of the trial objectives and group allocations. The statistician was also blinded to the group assignments. The CONSORT diagram is illustrated in Figure 1.

### 2.2. Participants

Sample size was determined using repeated-measures ANOVA for within–between interaction in G*Power (version 3.1.9.2). The analysis was based on two groups with two repeated measurements, a type I error rate of 0.05, and 80% power. Effect size estimation was based on partial eta-squared (η²p), with a medium effect size assumed (η²p = 0.06), consistent with recommendations by Lakens (2013) [54] and Espírito-Santo and Daniel (2018) [55], corresponding to a Cohen's f of 0.25. The correlation among repeated measures was set at 0.5, with a non-sphericity correction of ε = 1, as reported in previous trials recruiting volunteers with similar characteristics (i.e., overweight or obesity) and examining similar outcomes (29, 32, 34, 36, 39, 40). A total sample size of 56 participants (28 per group) was required. After considering a 20% dropout rate, a total of 70 participants (35 per group) were required.

This study included 69 patients who met the following inclusion criteria [56]: chronic knee pain for over three months, as well as at least three of the following: (i) age > 50 years, (ii) morning stiffness (<30 min), and (iii) crepitus and presence of osteophytes (mandatory). Additionally, all participants were diagnosed with grades 2 and 3 of KOA according to the Kellgren–Lawrence criteria, based on radiological assessments by a traumatologist or rheumatologist. The inclusion criteria also comprised individuals with T2DM with fasting plasma glucose levels exceeding 7.0 mmol/L and HbA1c levels above 6.5%, as well as those with a body mass index (BMI) of 25 kg/m² or above, currently receiving standard diabetes medications, and a negative result on a polymerase chain reaction (PCR) or rapid test for SARS-CoV-2.

Patients were excluded if they exhibited any of the following: (i) secondary KOA, (ii) acute knee pain, (iii) changes in medication, supplementation, or diet, (iv) changes in habitual physical activity, (v) intra-articular hyaluronic acid injection treatment within one year, (vi) smoking, (vii) dementia or any psychiatric diseases, (viii) less than 90% adherence to prescribed exercise sessions, or (ix) a positive SARS-CoV-2 test. They were advised to refrain from engaging in any other forms of exercise and to maintain their habitual physical activity levels and eating patterns throughout the three-month intervention period. The patients' logbooks were reviewed at each visit in order to ensure that there were no changes in nutritional behavior and physical activity. The non-exercising control group followed standard treatment (diabetes medications) without engaging in any form of structured exercise during the intervention. The participants' baseline characteristics are shown in Table 1.

**Table 1.** Participants' baseline characteristics.

Variable	CON (n = 35)	HBCT (n = 35)	*p-value*
Gender, n (%)			0.91
Male	16 (51.6)	15 (48.4)	
Female	19 (48.7)	20 (51.2)	
Age (years)	61.7 (5.2)	62.6 (6.9)	0.54
Weight (kg)	80.8 (15.9)	80.6 (10.4)	0.93
Height (m)	1.57 (0.86)	1.58 (0.88)	0.72
BMI (kg/m^2^)	32.8 (5.6)	32.4 (4.3)	0.88
Comorbidity score	6.1 (1.1)	5.70 (1.1)	0.37
T2DM medication, n (%)			0.87
Metformin	21 (52.5)	19 (47.5)	
Metformin and Gliclazide	12 (48.0)	13 (52.0)	
Actrapid	2 (40.0)	3 (60.0)	

Note: Values are expressed as mean (± SD). BMI, body mass index; T2DM, type 2 diabetes mellitus.

### 2.3. Exercise protocol

Patients were advised to maintain their usual physical activity levels throughout the course of the study. Any deviation from the established protocol was grounds for exclusion from the study. During the scheduled follow-ups, the participants were queried as to whether they had engaged in any activities that could be considered to interfere with the intervention. The exercise group performed HBCT on three occasions per week, on non-consecutive days, for a period of 12 weeks. The inaugural session was conducted at USM Hospital, during which participants were instructed in the proper form for the prescribed exercises. The HBCT protocol was used in a previously published study [53], aligning with the training recommendations previously documented [57]. Progressive overloading was implemented for the dual purposes of ensuring the safety of participants and facilitating continuous progress. Each session comprised seven exercises conducted in a circuit format. Two aerobic exercises were performed: the first was jogging in place, and the second was static cycling, with each exercise lasting one minute. Five muscle-strengthening exercises were also conducted: biceps curl, leg curl, lateral raise, scissor exercise, and standing chest fly. The exercises employed bodyweight movements and adjustable dumbbells to target all major muscle groups [58],[59]. Participants performed the prescribed repetitions with proper form and a moderate tempo for each exercise, maintaining a comfortable range of motion and avoiding any pain, while considering their existing physical limitations. During the initial six-week period, participants were instructed to perform 15 repetitions of each exercise, with two rounds completed in total. Each round was separated by 1 min of passive rest, and different exercises were performed with 1 min of rest between them. In weeks 7–12, the number of repetitions was gradually increased to 30 for 4 rounds, with the same rest intervals maintained. Each round lasted between 10 and 15 min, with total session durations ranging from 20 to 60 min. This allowed participants to gradually adapt to increased training volume. The objective of this training program was to prevent injuries related to training, overreaching, and overtraining while providing an engaging and inclusive exercise experience. To ensure that participants utilized the appropriate exercise techniques, a tutorial video was provided, demonstrating each exercise with proper form at a controlled, moderate pace. The tutorial video was made available following the inaugural session to assist participants in executing each exercise independently and without supervision.

In the initial phase of the intervention, patients were instructed to utilize a weight that would enable them to perform a specified number of repetitions without experiencing pain or discomfort. As the study progressed, they were then instructed to progress to heavier weights that would allow them to complete the prescribed number of repetitions at each exercise station. A 5-min warm-up and a 5-min cool-down period were implemented in all sessions. The rate of perceived exertion (RPE) was self-recorded using the Borg scale, which ranges from 6 (representing the level of exertion associated with rest) to 20 (representing maximal exertion) [60]. The RPE values for each round were recorded, and the mean exertion was subsequently calculated. Participants were counselled to adjust the magnitude of their exertion in a progressive manner throughout the 3-month intervention period. In particular, the following RPE ranges were recommended: weeks 1–6, RPE 11–13; weeks 7–12, RPE 14–16. Participants' exercise adherence and RPE values per session were recorded in a logbook. The logbook was reviewed on a weekly basis via telephone calls and during each visit. Furthermore, the HBCT group received a weekly text message from the researchers, which addressed queries pertaining to the exercise program.

### 2.4. Assessment procedures

All patients were advised to refrain from consuming caffeinated beverages and engaging in strenuous exercise for a period of 24 h prior to their initial visit. All outcomes were evaluated at each visit (pre-, mid-, and post-training) in morning hours (7:00–9:00 a.m.) after an overnight fast. All assessment procedures took place at USM Hospital. At the first visit (pre-training), assessments included cardiovascular parameters (e.g., resting heart rate and systolic and diastolic blood pressure), BMI, biomarkers (e.g., HbA1c, interleukin 6, and superoxide dismutase), rate of perceived exertion, blood oxygen levels, and a 6-min walk test to evaluate cardiorespiratory fitness (6MWT). Additionally, KOA symptoms and comorbidity scores were assessed. At the second visit (mid-training: 6-week follow-up), cardiovascular parameters, KOA symptoms, rate of perceived exertion, blood oxygen levels, and the 6-min walk test were evaluated. At the third visit (post-training: 12-week follow-up), the same measurements as those conducted at the first visit were performed.

#### 2.4.1. Resting cardiovascular function

A physician measured resting heart rate (RHR) and peripheral oxygen saturation (SpO_2_) using a pulse oximeter (Choicemmed Oxywatch MD300C11, Beijing, China). Blood pressure was assessed with an arm sphygmomanometer and stethoscope kit (Tanaka 500-W-2, Sangyo Co., Ltd., Tokyo, Japan) following standard procedures. Specifically, blood pressure was measured twice in a seated position on both arms, with a 1-min interval between measurements. No significant differences were observed between arms, and the median of all measurements was reported as the blood pressure value [49].

#### 2.4.2. Biomarkers

Blood samples and assays were conducted following established protocols as previously outlined [49],[61]. Specifically, 10 mL blood samples were collected from participants after a 12-h fast, placed in heparinized sterile tubes, and kept on ice until processing. The blood was centrifuged at 1500 rpm for 15 min at 4 °C. The separated serum was stored at −80 °C until further analysis. Blood samples for HbA1c and renal function parameters (including potassium, sodium, total bilirubin, and urea) were immediately sent to the endocrine laboratory for analysis. Plasma interleukin-6 (IL-6) and superoxide dismutase (SOD) levels were measured by enzyme immunoassay using Human ELISA kit #P05231 and # P00441, respectively. The inter- and intra-assay coefficients of variability for all assays ranged from 5.2% to 10.1% and from 4.8% to 9.5%, respectively.

#### 2.4.3. Cardiorespiratory fitness

The 6MWT was carried out in a straight hallway in accordance with the American Thoracic Society guidelines. Ten minutes before the test, the patient received a standard instruction and was instructed to rate their level of breathlessness using the modified Borg Dyspnea Scale (CR–10) [62]. The patient's RHR and blood pressure were measured before the test. The patient did not receive direction or encouragement during the test. The test was not paused when patients stopped during the test. The test was stopped if i) the patient requested it or ii) there was severe dyspnea, chest discomfort, leg cramps, staggering, or diaphoresis. All aforementioned parameters were also evaluated immediately after the test. In accordance with the recommendations of the American Thoracic Society, we did not check SpO_2_ throughout the test [63].

#### 2.4.4. Knee osteoarthritis-related health outcomes and comorbidity evaluation

The assessment of KOA symptoms was conducted using the validated Malay version of the knee injury and osteoarthritis outcome score (KOOS) questionnaire. The KOOS is self-administered and assesses five health outcomes: pain (KOOS-pain), symptoms (KOOS-symptom), activities of daily living (KOOS-ADL), sport and recreation function (KOOS-sport), and knee-related quality of life (KOOS-QoL) for patients with KOA on a Likert scale from zero to four (worst to best) [64].

The Charlson Comorbidity Score (CCS) was used to evaluate patients' health status, examining 17 chronic diseases with assigned weighted scores or index values [65]. Patients' CCS was used to group the comorbidity into the following groups: 0, None; 1–2, Mild; 3–4, Moderate; and ≥5, Severe [66],[67].

### 2.5. Statistical analysis

The analysis started with an exploration of the data to assess normality via Shapiro–Wilk tests and identify any potential errors in data entry. Participants' baseline characteristics (Table 1) were compared using an independent t-test for normally distributed data and the Mann–Whitney U test for non-normally distributed data. To compare groups at each time point, a 2 × 2 (group × time) repeated-measures ANOVA with a Bonferroni post-hoc analysis was utilized. The Greenhouse–Geisser correction was applied if the assumption of sphericity was violated. Pre-intervention values were compared with data recorded 12 weeks post-intervention within and between groups using repeated-measures ANOVA. A p-value of less than 0.05 was considered statistically significant. Statistical analysis was conducted using SPSS 27.0 software (IBM Corp., Armonk, NY, USA).

## 3. Results

Of 1033 patients screened for eligibility, 963 (93%) were excluded. Of these, 934 (96%) did not meet the inclusion criteria, while 26 (3%) declined to participate. The remaining three exclusions (0.3%) were for other reasons, such as family and work commitments. Therefore, a total of 69 participants (HBCT, n = 34; CON, n = 35) completed the study, and the participation rate for HBCT was 91.7% (Figure 1). The adherence rate was 98.6%, with one patient in the HBCT group withdrawing due to loss to follow-up (dropout rate: 1.4%), whereas patients in CON attended all three visits. No injuries or adverse events were reported during the intervention. No changes in habitual physical activity, dietary habits, CCS score, or medication use were observed during the study.

**Table 2.** Baseline comparisons.

Variable	CON (n = 35)	HBCT (n = 34)	*p-value*
SBP (mmHg)	139.34 ± 18.25	142.44 ± 14.45	0.44
DBP (mmHg)	81.23 ± 8.32	80.50 ± 8.83	0.37
BMI (kg/m^2^)	32.81 ± 5.59	32.41 ± 4.25	0.73
6MWT (m)	265.71 ± 78.90	230.0 ± 42.14	0.02*
RPE (0–10)	7.86 ± 2.12	8.85 ± 1.48	0.43
RHR (bpm)	78.71 ± 14.30	77.70 ± 8.72	0.72
SpO_2_ (%)	95.23 ± 2.04	95.09 ± 1.42	0.74
HbA1c (%)	7.23 ± 0.63	7.34 ± 0.53	0.40
IL-6 (pg/mL)	268.86 ± 77.15	269.12 ± 44.25	0.42
SOD (ng/mL)	11.11 ± 3.80	10.26 ± 2.87	0.52
Potassium (mmol/L)	4.43 ± 0.51	4.25 ± 0.50	0.15
Sodium (mmol/L)	138.43 ± 2.60	138.44 ± 2.52	0.98
Total bilirubin (µmol/L)	11.17 ± 3.92	10.71 ± 5.25	0.68
Urea (mmol/L)	5.58 ± 1.79	4.77 ± 1.79	0.07
KOOS-QoL	41.91± 24.66	49.79 ± 21.46	0.16
KOOS-ADL	68.17 ± 20.22	68.85 ± 21.42	0.89
KOOS-sport	36.0 ± 25.40	32.65± 25.08	0.93
KOOS-pain	66.43 ± 17.84	67.65 ± 14.05	0.75
KOOS-symptom	66.66 ± 19.13	69.18 ± 15.25	0.55

Note: Values are expressed as mean ± SD. BMI, body mass index; RHR, resting heart rate; SBP, systolic blood pressure; DBP, diastolic blood pressure; SOD, superoxide dismutase; IL-6, interleukin-6; HbA1c, glycated hemoglobin; SpO2, blood oxygen levels; 6MWT, 6-min walk test; QoL, quality of life; ADL, activities of daily living. *: denotes statistical significance (p < 0.05).

**Table 3.** Group-by-time interaction effects.

Variables	Time	MD (95% CI)	*p-value*
SPB (mmHg)	Pre	3.10 (11.02, −4.82)	0.44
	Mid	−7.24 (−14.61, 0.13)	0.05
	Post	−14.20 (−21.86, −6.54)	<0.001*
DBP (mmHg)	Pre	−0.73 (−4.85, 3.39)	0.73
	Mid	−5.47 (−9.29, −1.66)	0.006*
	Post	−10.40 (−14.33, −6.47)	<0.001*
BMI (kg/m^2^)	Pre	−0.40 (−2.79, 1.99)	0.74
	Mid	−1.14 (−3.73, 1.46)	0.39
	Post	−2.47 (−4.88, −0.06)	0.05*
6MWT (m)	Pre	−35.71 (−66.24, −5.19)	0.02*
	Mid	3.37 (−27.60, 34.34)	0.83
	Post	37.06 (6.267, 67.86)	0.02*
SpO_2_ (%)	Pre	0.14 (−0.70, 0.99)	0.47
	Mid	−0.33 (−1.53, 0.87)	0.58
	Post	−2.33 (−3.07, −1.59)	<0.001*
RPE (0–10)	Pre	−0.10 (−1.87, −0.12)	0.03*
	Mid	−0.38 (−0.47, 1.23)	0.38
	Post	−1.38 (−2.14, −0.62)	<0.001*
HbA1c (%)	Pre	0.12 (−.162, 0.40)	0.41
	Post	−0.65 (−1.003, −0.30)	<0.001*
IL-6 (pg/mL)	Pre	0.85 (0.77, 2.47)	0.30
	Post	1.72 (0.147, 3.29)	0.03*
SOD (ng/mL)	Pre	0.85 (0.77, 2.47)	0.300
	Post	1.72* (0.147, 3.29)	0.033
KOOS-QoL	Pre	−7.88 (−19.0, 3.24)	0.16
	Mid	−14.26 (−24.39, −4.13)	0.007*
	Post	−17.84 (−26.16, −9.52)	<0.001*
KOOS-symptom	Pre	−2.52 (−10.85, 5.81)	0.55
	Mid	−5.11 (−12.38, 2.17)	0.17
	Post	−11.74 (−18.03, −5.44)	<0.001*
KOOS-pain	Pre	−1.22 (−8.95, 6.51)	0.75
	Mid	−5.28 (−12.38, 1.812)	0.14
	Post	−18.60 (−25.95, −11.25)	<0.001*
KOOS-ADL	Pre	−0.68 (−19.0, 9.32)	0.89
	Mid	−12.0 (−21.24, −2.77)	0.01*
	Post	−15.77 (−23.31, −8.22)	<0.001*
Potassium	Pre	−0.18 (−.423, 0.07)	0.15
	Post	0.53 (0.10, 0.97)	0.02*

Note: Values are expressed as mean differences (95% confidence intervals). BMI, body mass index; RHR, resting heart rate; SBP, systolic blood pressure; DBP, diastolic blood pressure; SOD, superoxide dismutase; IL-6, interleukin-6; HbA1c, glycated hemoglobin; SpO2, blood oxygen levels; 6MWT, 6-min walk test; QoL, quality of life; ADL, activities of daily living. *: denotes statistical significance (p < 0.05).

At baseline, there was no significant difference between HBCT and CON in SBP, DBP, BMI, RPE, heart rate, SPO2, HbA1c, IL-6, SOD, potassium, sodium, total bilirubin, urea, KOOS-QoL, KOOS-ADL, KOOS-sport, KOOS-pain, and KOOS-symptom. During the 6MWT, there was a significant difference between HBCT and CON, since CON significantly walked a greater distance than HBCT (*p* = 0.02) (Table 2).

At post-training, significant changes were found in SBP, DBP, HbA1c, IL-6, SOD, potassium (*p* < 0.001), and BMI (*p* = 0.05) between CON and HBCT. At mid-training, a significant difference was detected only in DPB between CON and HBCT (*p* = 0.006). Regarding 6MWT and RPE, meaningful differences were found between CON and HBCT at baseline (*p* = 0.02) and post-training (*p* = 0.02). In terms of KOOS parameters, substantial improvements were observed between CON and HBCT in KOOS-QoL and KOOS-ADL at mid- (*p* = 0.007) and post-training (*p* < 0.001), whereas KOOS-pain and KOOS-symptom showed significant alterations only at post-training (*p* < 0.001). Table 3 presents the group-by-time interaction effects in detail.

Table 4 shows the comparison within groups over time. For HBCT, significant reductions were observed in SBP (−6.2%), DBP (−5%), and BMI (−3.9%) for HBCT at mid-training (*p* = 0.001). Similar improvements were detected in SBP (−9.5%), DBP (−9.3%), and BMI (−5.1%) for HBCT compared to CON at post-training (*p* = 0.001). However, there were no significant differences in SPB, DBP, and BMI from mid- to post-training for HBCT. For CON, there was no significant difference in SPB, DBP, and BMI throughout the intervention.

HBCT demonstrated a significant increase in 6MWT (pre vs. mid: 16.5%; pre vs. post: 23.3%; mid vs. post: 12.7%; *p* < 0.001) and RPE (pre vs. mid: 6%; pre vs. post: 29.2%; mid vs. post: 17.3%; *p* < 0.001). CON showed no significant differences in 6MWT and RPE throughout the intervention.

As for the biomarkers, the pairwise comparisons in time demonstrated significant alterations in HbA1c (−9.1%, *p* < 0.001), IL-6 (−15%, *p* < 0.001), potassium (−16.7%, *p* < 0.001), and SOD (+17.5%, *p* = 0.004) in HBCT, but not in CON.

In terms of KOOS, HBCT elicited beneficial changes (*p* = 0.042) in KOOS-QoL (18.8%), KOOS-symptom (10.3%), and KOOS-pain (16.6%) scores at mid-training. Also, HBCT conveyed significant improvements (*p* < 0.001) in KOOS-QoL (31.2%), KOOS-symptom (16.8%), and KOOS-pain (19.8%) scores at post-training. CON did not exhibit any alterations in all KOOS parameters over time. Other parameters, including potassium, sodium, total bilirubin, urea, and KOOS-sport, did not demonstrate significant interactions between time and treatment (data are not shown in Tables 3 and 4).

**Table 4.** Pairwise comparisons between HBCT and CON in time.

Variables	CON						HBCT					

	Pre vs. Mid (95% CI)	*p*	Pre vs. Post (95% CI)	*p*	Mid vs. Post (95% CI)	*p*	Pre vs. Mid (95% CI)	*p*	Pre vs. Post	*p*	Mid vs. Post	*p*
SBP (mmHg)	−1.54 (−7.11, 4.02)	0.95	−3.74 (−9.71, 2.23)	0.37	−2.20 (−8.94, 4.53)	0.09	8.79* (3.19, 14.30)	0.001	13.56* (8.09, 19.03)	<0.01	4.77 (−2.40, 11.92)	0.31
DBP (mmHg)	−0. 69 (−2.86, 15)	0.95	−2.17 (−6.07, 1.72)	0.51	−1.49 (−4.72, 2.86)	0.95	4.06* (1.55, 6.57)	0.001	7.50* (4.03, 10.97)	<0.01	3.44 (−0.15, 7.03)	0.06
BMI (kg/m^2^)	−0.28 (−0.91, 0.35)	0.81	−0.30 (−0.75, 0.15)	0.30	−0.02 (−0.57, 0.52)	0.95	1.25 (−1.02, 3.51)	0.002	1.65* (1.25, 2.05)	<0.001	0.405 (−1.943, 2.752)	0.60
6MWT (m)	1.14 (−4.47, 6.75)	0.95	5.57 (−8.18, 19.32)	0.94	4.43 (−8.27, 17.12)	0.95	37.94* (−48.83, −27.05)	<0.001	67.21* (−86.87, −47.54)	<0.001	29.27* (−46.40, 12.13)	<0.001
SpO_2_ (%)	−0.11 (−0.91, 0.69)	0.95	0.03 (−0.69, 0.91)	0.95	0.14 (−0.90, 1.18)	0.95	−0.59 (−1.94, 0.77)	0.85	−2.44* (−3.20, −1.68)	<0.001	−1.85* (−3.09, −0.62)	0.002
RPE (0–10)	−0.09 (−0.45, 0.28)	0.95	−0.31 (−0.79, 0.16)	0.31	−0.23 (−0.77, 0.45)	0.85	0.53* (0.11, 0.95)	0.009	2.06* (1.55, 2.57)	<0.001	1.53* (0.99, 2.06)	<0.001
HbA1c (%)	-	-	−0.10 (−0.39, 0.19)	0.50	-	-	-	-	0.67* (0.40, 0.94)	<0.001	-	-
IL-6 (pg/mL)	-	-	2.29 (−4.68, 9.25)	0.51	-	-	-	-	35.15* (25.57, 44.73)	0.001	-	-
SOD (ng/mL)	-	-	0.77 (−0.53, 2.07)	0.24	-	-	-	-	1.794* (0.61, 2.97)	0.004	-	-
K (mmol/L)	-	-	1.78 (−0.40, 0.40)	0.95	-		-	-	−0.71* (−0.99, −0.43)	<0.001	-	-
KOOS-QoL	−1.80 (−7.81, 4.21)	0.95	−5.60 (−15.03, 3.83)	0.43	−3.80 (−11.01, 3.41)	0.58	−8.18 (−18.08, 1.73)	0.14	−15.56* (−23.40, −7.71)	<0.001	9.38 (−1.33, 16.09)	0.042
KOOS-symptom	−1.97 (−7.22, 3.28)	0.95	−2.43 (−9.70, 4.84)	0.95	−0.46 (−7.34, 6.42)	0.75	−4.56 (−11.74, 2.62)	0.36	−11.65* (−18.66. −4.62)	0.001	−7.09* (−10.16, −4.02)	<0.001
KOOS-pain	1.86 (−3.20, 6.91)	0.85	4.00 (−4.74, 12.74)	0.77	2.14 (−5.16, 9.45)	0.95	−2.21 (−6.54, −2.13)	0.62	−13.38* (−18.77. −7.98)	<0.001	−11.18* (−16.68, −5.67)	<0.001
KOOS-ADL	−0.97 (−7.75, 5.80)	0.95	−2.89 (−9.20, 3.42)	0.77	−1.91 (−7.42, 3.59)	0.95	−12.29* (−20.08, −4.51)	<0.001	−17.97* (−26.82 −126)	<0.001	−5.68 (−14.04, 2.69)	0.29

Note: Values are expressed as mean difference (95% confidence intervals). ADL, activities of daily living; BMI, body mass index; DBP, diastolic blood pressure; HbA1c, glycated hemoglobin; IL-6, interleukin-6; K, potassium; QoL, quality of life; RHR, resting heart rate; SBP, systolic blood pressure; SOD, superoxide dismutases; SpO_2,_ blood oxygen levels6MWT, 6-min walk test; *: denotes statistical significance (p < 0.05).

## 4. Discussion

This study aimed to evaluate the effectiveness of a 12-week HBCT on several indices related to KOA and cardiometabolic health among older adults with KOA and diabesity. To the best of our knowledge, this is the first randomized controlled trial examining the effects of such a home-based protocol in this population, using bodyweight training that has been reported as a popular exercise mode [68],[69]. The present findings showed that the exercise protocol was safe for this population and substantially improved anthropometric characteristics, resting cardiovascular function, glucose metabolism, aerobic capacity, and KOA parameters related to quality of life and functionality. These results underscore the importance of implementing combined cardiorespiratory and muscle-strengthening activities in a circuit fashion in a free-living environment, suggesting real-world exercise strategies for cohorts of significant importance.

### 4.1. Glycemic control

The improved glycemic control observed in this study is consistent with previous studies [70]–[72], suggesting that HBCT may increase insulin sensitivity, which contributes to managing T2DM by enabling cells to uptake blood glucose for energy. A low HbA1c level may be caused by the fact that during exercise, glucose is also absorbed from the bloodstream and delivered to the working muscles and organs. A meta-analysis of randomized, controlled trials revealed a significant reduction in HbA1c and basal blood glucose after six months of commencement of physical activity [73]. When combined aerobic and resistance training was compared with controls, previous studies have shown a significant reduction in HbA1c and a favorable trend for glycemia [71],[72]. Interestingly, a systematic review of 37 trials involving 2208 adults with T2DM reported a substantially lowered HbA1c in a combined aerobic and resistance training group compared to the non-exercise group [74]. Given that a 1% absolute reduction in HbA1c levels has been associated with a 15%–20% reduction in major CVD events and a 37% reduction in microvascular complications [75], a reduction in HbA1c can have significant health implications. Regular exercise has been reported to reduce intramyocytic lipid concentration and enhance fatty acid oxidation ability, which could explain these positive findings [76],[77]. The combination training group experienced a greater activation of muscles in both the upper and lower limbs during exercise. The overall intramyocytic fat content is probably reduced and/or the fatty acid oxidation capacity is enhanced more by combination training, leading to improved glucose absorption after the combination training, since the combination program comprised a larger muscle mass compared to either aerobic or resistance training alone [78],[79]. In fact, Sigal et al. (2007) reported improved HbA1c levels following combined aerobic and resistance training, but not after aerobic or resistance training alone [80]. It seems that to attain glycemic control, intensive exercise programs are required, since such exercise approaches, including circuit training, appear to be an alternative resistance-based training solution that can be less physically challenging and more engaging [81], but still efficient for improving muscular strength, aerobic fitness, body composition, and CVD risk factors [80],[82].

### 4.2. Inflammation

Importantly, IL-6 levels significantly decreased after 12 weeks of HBCT compared to controls in the present study. This outcome may suggest that such an exercise program is beneficial for lowering chronic systemic inflammation commonly observed among older adults with diabesity. This observation may be supported by the fact that BMI was lower post-training, which is a factor associated with the decline of pro-inflammatory markers in populations with excess body weight and impaired metabolic health [83]. Additionally, we found a significant increase in SOD following the intervention compared to the non-exercising group, which may be attributed to the established link between insulin resistance and increased oxidative stress, as well as impaired antioxidant defense systems. By improving insulin sensitivity, exercise can help reduce oxidative stress and enhance SOD activity [84], which is critical as diabesity is associated with chronic low-grade inflammation that can contribute to oxidative stress. Exercise has been shown to reduce inflammation markers, such as IL-6 and pro-inflammatory cytokines. By reducing inflammation, exercise may indirectly improve SOD activity [85]. Also, the IL-6 concentration was very high in both CON and HBCT (>260 pg/mL), which is usually found in acute inflammation instead of chronic inflammatory diseases in older adults [86]. Such an observation may be explained by the increased psychological stress levels [87] that potentially occurred among participants with comorbidities [88].

### 4.3. Anthropometry

In this study, BMI was significantly improved. This finding could be attributed to circuit training that combines aerobic and resistance sequences of exercises with short rest intervals, as well as high adherence to the exercise protocol throughout the study [53]. It is noteworthy that a training type integrating body-weight resistance exercises and adjunct modalities into a single workout regimen may prove effective in promoting favorable changes in anthropometrics, attributable to their substantial energy expenditure [89],[90]. This result is in line with previous studies investigating circuit-based exercise regimens that reported high compliance with prescribed exercise interventions [42],[45],[47],[49],[59],[91],[92]. In contrast, another study did not find any significant differences in BMI among T2DM patients [93].

### 4.4. Resting cardiovascular function

As for the resting cardiovascular function, this pragmatic trial detected meaningful reductions in SBP and DBP in HBCT, but not in CON. These improvements may reflect the adherence to the exercise protocol while being linked to the positive changes in both BMI and HbA1c. This result is aligned with previous studies examining the effectiveness of combined aerobic and resistance training [91],[94]. However, another study found no blood pressure reductions following a high-intensity resistance training program among T2DM patients [95], suggesting that lower exercise intensities may be more beneficial for BP. Our study, however, showed no beneficial changes in RHR. Given that RHR is directly controlled by the autonomic nervous system, with parasympathetic vagal activity predominating during rest and gradually decreasing as exercise progresses, further research is needed in this direction. Similarly, Yavari, Najafipoor [96] reported no meaningful changes in RHR among T2DM patients following aerobic, resistance, or combined training. In general, it is not clear whether CT can lower RHR among people with metabolic health impairments.

### 4.5. Physical function

Notably, T2DM patients are more likely to demonstrate poor muscular fitness [97] and lower leg skeletal mass than those without diabetes [98]. The impaired muscular function in patients with T2DM may result in insulin resistance [99]. A primary rationale for patients afflicted with KOA who experience severe, acute, or chronic symptoms to engage in physical activity is the enhancement of their physical functionality [100]. Physical function and functional capacity may be evaluated using patient-reported or performance-based measures, regarded as a required outcome in all KOA clinical studies [101]. The 6MWT is a performance-based test that measures submaximal aerobic capacity and long-distance walking ability in patients with KOA and is recommended by the Osteoarthritis Research Society International [102]. In our study, we found meaningful improvements in 6MWT, as well as higher RPE and SpO_2_ following the 12-week intervention, corroborating a previous study [103]. The improvement in walking distance suggests that HBCT enhances muscular strength and mobility of the knee joint in patients with KOA. Noteworthy, CON showed lower walking performance at post-testing compared to baseline, which has also been reported previously [104] and may be attributed to prolonged inactivity that attenuates functionality among people with musculoskeletal health impairments. Moreover, the SpO_2_ levels were significantly improved in HBCT. According to Garg, Gupta [105], obesity is an independent contributor to the decrease in SpO_2_ in T2MD patients and a crucial factor in the development of diabetic complications regarding tissue hypoxia. A previous study postulated that by enhancing circulation at the extremities, exercise might alleviate chronic hypoxia-related micro- and macrovascular alterations in the tissues and organs of T2DM patients [106]. We also found significant improvements in RPE following the intervention, suggesting that RPE may be an effective instrument for T2DM patients in order to control the intensity when engaging in physical activity, which leads to better outcomes, a lower risk of injury, and a higher desire to exercise [107],[108].

### 4.6. Knee osteoarthritis-related health outcomes

In the present study, we also found significant improvement in KOOS-QoL, KOOS-ADL, KOOS-pain, and KOOS-symptom, suggesting that living an active lifestyle leads to better outcomes in happiness, health, relationships, and overall life satisfaction. These positive changes in KOOS-related outcomes are in line with the main findings reported by Skou, Rasmussen [109], indicating similar benefits in various KOOS parameters among patients with KOA following a 12-week CT program compared to those following usual care and having few treatment-related adverse events. The beneficial alterations in KOA-related measurements observed here are important given the strong association of KOA with inactivity and impaired metabolic health that characterized participants in the present trial. Notably, the decrease in IL-6 may suggest lower systemic inflammation, leading to a decrease in inflammatory pain and partially explaining the significant improvement in KOOS pain and high exercise compliance [110]. Such improvements highlight the vital role of structured exercise training in physical and mental health among patients with KOA and diabesity.

Individuals with diabesity and musculoskeletal health issues show poor quality of life due to physical limitations and impaired mental health [111]. In the present study, we found a significant improvement in quality of life, suggesting that HBCT may mitigate the patients' functional deficits and elevate health-related quality of life. The substantial increase in walking ability in those who completed the 12-week HBCT protocol may be a critical factor from both a physiological and psychological standpoint [112]. This observation is supported by a similar study showing that a 16-week exercise intervention improved both walking capacity and quality of life in older adults with T2DM [113]. Similarly, Reid, Tulloch [114] found significant improvements in quality of life following aerobic, resistance, or combined training compared to a non-exercising group. In addition, a decrease in all-cause mortality, including CVD, may be attributed to the increased walking ability. However, previous studies investigating quality of life among T2DM patients reported no significant differences between combined aerobic and resistance training and controls [104].

### 4.7. Public health implications

Circuit training demonstrates clinically meaningful benefits for older adults with KOA and metabolic dysregulation by simultaneously improving cardiovascular risk profiles, physical performance, and health-related quality of life. Notably, some discrepancies with earlier osteoarthritis literature—where quality-of-life improvements are inconsistently reported—may be explained by the multimodal and progressive nature of circuit training, which addresses both pain-related disability and metabolic dysfunction more comprehensively than single-mode exercise [115]. From a public policy perspective, these results support the inclusion of structured, home-based circuit training within chronic disease and healthy ageing strategies to overcome barriers related to cost, access, and mobility. For the purpose of practical application, circuit training represents a pragmatic, adaptable intervention capable of targeting cardiometabolic risk and functional decline concurrently, reinforcing its value in integrated care models for complex older populations [116]. In summary, while single exercise modes remain beneficial, circuit training offers a promising strategy to reduce cardiovascular risk and improve body functionality and quality of life in older adults with diabesity and KOA. Further research is needed to optimize its prescription and implementation across clinical settings.

### 4.8. Limitations

The generalizability of these findings is limited by the restricted age range, BMI, and racial composition of the sample. Reliance on the KOOS alone may have underestimated fluctuations in disease activity between assessments, while the small sample size, single-center design, and potential self-care bias further constrain interpretation. The absence of session-specific pain monitoring and exercise enjoyment measures limits insight into training tolerance, adherence mechanisms, and psychophysiological responses, despite high adherence and attendance rates. Although the 6MWT is a valid functional measure, a maximal cardiopulmonary exercise test would have provided a more robust assessment of cardiorespiratory fitness. Additionally, the fixed weekly training frequency precluded evaluation of dose–response effects, and the lack of comparison with other exercise modalities limits conclusions regarding the relative efficacy of home-based circuit training [117]. Finally, exclusive reliance on self-reported physical activity and dietary data introduces potential reporting bias, underscoring the need for objective monitoring in future studies.

## 5. Conclusion

The present study showed positive effects of an HBCT program on CVD risk factors, functionality, quality of life, and CRF among older adult patients with KOA and diabesity. The present results are important since these populations are characterized by cardiovascular, metabolic, and musculoskeletal impairments, resulting in an increased risk of developing obesity-related comorbidities. These findings have public health implications as they recommend a feasible and injury-free exercise approach incorporating muscle strengthening–based activities into a circuit-type program, which affects numerous health and fitness parameters in a home-based setting. Taking these results into account, clinicians and practitioners may have an additional real-world exercise option for sedentary individuals with an unhealthy BMI and cardiometabolic dysfunction.

## Use of AI tools declaration

The authors declare they have not used Artificial Intelligence (AI) tools in the creation of this article.
